# Increasing health equity and access to skilled birth delivery services for the poor through community-based health-care interventions: evidence from northern Nigeria

**DOI:** 10.1186/1472-6963-14-S2-P90

**Published:** 2014-07-07

**Authors:** Ekechi Okereke, Ibrahim Yisa, Adekunle Akerele, Benson Obonyo, Mike Egboh

**Affiliations:** 1 Abt Associates Nigeria/Partnership for Transforming Health Systems Phase 2, Abuja, Federal Capital Territory, Nigeria

## Background

The Partnership for Transforming Health Systems Phase 2 (PATHS2) project introduced and supports community based interventions such as safe motherhood initiative demand side (SMID) and an emergency transport scheme within northern Nigeria. This study evaluates the effect of PATHS2’s community based interventions in addressing transportation as a barrier to accessing skilled birth delivery services in health facilities.

## Methods

The PATHS2 project conducted baseline and midline surveys in 2009 and 2012 respectively to evaluate the impact of its health care interventions. Structured questionnaires were applied to respondents in randomly selected households within PATHS2’s intervention communities in Kano and Jigawa States during the baseline and midline surveys. The study respondents were categorized into five wealth quintiles using principal component analysis. Data analysis was undertaken using SPSS version 20.

## Results

Within intervention communities in Kano and Jigawa States, the proportion of respondents in the poorest and second poorest wealth quintiles at baseline relative to midline who indicated that transportation is a barrier to accessing skilled birth delivery services decreased by 28% and 24% (p<0.001) for Kano State as well as 21% and 25% (p<0.001) for Jigawa State respectively. There were no significant decreases in the proportion of respondents who indicated that transportation is a barrier to accessing skilled birth delivery services among the middle to richest wealth quintiles in both States. Within rural intervention communities in Kano State, the proportion of respondents at baseline relative to midline who indicated that transportation is a barrier to accessing skilled birth delivery services decreased by 11.9% (p<0.001). According to multivariate logistic regression analysis, respondents within intervention communities in the poorest wealth quintiles (p=0.023; OR 3.40, 95% CI: 1.18-9.79) and second poorest wealth quintiles (p=0.019; OR 3.51, 95% CI: 1.23-10.10) in Jigawa State are about four times less likely to indicate that transportation is a barrier to accessing skilled birth delivery services. While respondents from rural intervention communities in Kano State are four times less likely to indicate that transportation is a barrier to accessing skilled birth delivery services (p=0.027; OR 3.80, 95% CI: 1.16-12.49) following the implementation of PATHS2’s community based interventions.

## Conclusions

These findings demonstrate that PATHS2 is addressing the ‘transportation challenge’ as a barrier to accessing skilled birth delivery services, particularly for the poor. The results from PATHS2’s end-line survey in 2014 are expected to substantiate this evidence more strongly.

